# Whole-body tomographic fluorescence lifetime imaging of PD-L1 expression

**DOI:** 10.21203/rs.3.rs-9682833/v1

**Published:** 2026-06-03

**Authors:** Rahul Pal, Murali Krishnamoorthy, Satoru Morita, Atsuyo Morita, Asemare M. Taddese, Hajime Taniguchi, Xin Liu, Dan G. Duda, Anand T.N. Kumar

**Affiliations:** 1 Department of Otolaryngology-Head and Neck Surgery, Massachusetts Eye and Ear, Harvard Medical School, Boston, Massachusetts; 2 Department of Medical Sciences & Technology, Indian Institute of Technology Madras, Chennai, Tamil Nadu-600036, India; 3 Edwin. L. Steele Laboratories for Tumor Biology, Department of Radiation Oncology, Massachusetts General Hospital and Harvard Medical School, Boston, Massachusetts; 4 Department of Surgery, Keio University School of Medicine, Tokyo, Japan; 5 Department of Dermatology, University of Tokyo Hospital, Tokyo, Japan; 6 Transplant Oncology and Therapeutics Program, Department of Surgery, J.C. Walter Jr. Transplant Center, Houston Methodist Research Institute, Houston, Texas

## Abstract

Noninvasive quantification of immune markers such as programmed death ligand-1 (PD-L1) remains a major challenge because conventional molecular imaging methods cannot readily distinguish target-bound from nonspecifically retained probes within the tumor microenvironment. Here, we establish a framework for noninvasive quantification of PD-L1 expression in deep-seated hepatocellular carcinoma (HCC) using asymptotic time-domain (ATD) fluorescence lifetime tomography. *In vivo* time-domain fluorescence imaging was performed in mice bearing orthotopic HCC tumors following administration of a PD-L1-targeted near-infrared fluorescent probe (αPDL1-800). Multi-exponential analysis of time-domain fluorescence data was used to derive four amplitude-based metrics of PD-L1 expression. Quantitative *in vivo* measurements obtained from ATD tomography were validated against *ex vivo* PD-L1 expression measured by Western blotting. Among the evaluated metrics, a normalized parameter that accounts for inter-lifetime crosstalk demonstrated the strongest correlation with *ex vivo* PD-L1 expression (r^2^ = 0.77). These findings establish a foundation for a noninvasive, nonionizing imaging approach to quantify and monitor receptor expression and support a future path for the longitudinal assessment of immune biomarkers in both preclinical studies and clinical settings.

## Introduction

Immune checkpoint blockade (ICB) targeting the PD-1/PD-L1 axis has revolutionized cancer therapy, offering durable responses across diverse malignancies including non-small cell lung cancer, melanoma, and hepatocellular carcinoma (HCC)^1–5^. PD-L1 expression on tumor and immune cells has emerged as a clinically actionable biomarker that guides treatment selection and predicts therapeutic efficacy^6–9^. In HCC, multiple ICB regimens involving PD-L1 blockade, such as the combination of atezolizumab plus bevacizumab, and durvalumab plus tremelimumab, have gained FDA approval as first-line therapies for advanced disease^10–12^. These advances highlight the importance of understanding PD-L1 biology in HCC and its potential as a biomarker for predicting response and resistance to ICB therapy. However, the clinical utility of PD-L1 as a biomarker in HCC is limited as its expression is heterogeneous and subject to dynamic regulation by microenvironmental cues and therapeutic pressure^13–16^. For instance, PD-L1 levels can shift significantly following neoadjuvant chemotherapy or radiotherapy, transforming an immune-resistant tumor microenvironment (TME) into one more receptive to immunotherapy^17–19^. Moreover, conventional techniques such as immunohistochemistry (IHC) and flow cytometry provide only static measurements from small tumor regions^20^. These limitations hinder the use of conventional histological techniques for dynamic monitoring of PD-L1 expression and longitudinal monitoring of therapy responses within the same subject^21^.

Noninvasive molecular imaging using nuclear tracers or optical probes presents an attractive approach for *in vivo* assessment of PD-L1 expression. Several PD-L1 targeted PET and fluorescent contrast agents have been developed and evaluated in both preclinical models and clinical studies^22–25^. PET imaging has demonstrated the potential to measure PD-L1 levels and monitor immunotherapy response in patients^26,27^. However, repeated PET scans pose challenges due to cumulative radiation exposure, particularly in an already vulnerable patient population^28,29^. Furthermore, high cost and limited accessibility can constrain its use for frequent longitudinal monitoring. Several optical imaging approaches employing near-infrared (NIR) fluorescently labeled anti-PD-L1 antibodies have demonstrated feasibility for measuring PD-L1 expression in superficial xenograft tumors^22,30,31^. While whole-body PET imaging has been evaluated in humans, fluorescence imaging offers a complementary, low cost, nonionizing approach for longitudinal studies in preclinical models and in select human cancers, including oral, skin and breast cancer^32–34^. Tumor targeted fluorescent probes also enable *in vivo* whole-animal optical tomography in preclinical models, offering a powerful tool to visualize three-dimensional probe distribution and provide molecular information that reflects target availability and therapy response^35–38^.

*In vivo* molecular imaging using PET or optical contrast agents typically involves measuring total signal intensity to assess tumor contrast or estimate probe uptake in tumors^39,40^. However, biomarker targeted probes frequently exhibit prolonged retention times in off-target organs (e.g., liver, kidney, and spleen), resulting in poor tumor-to-background signal ratio^41^. Additionally, the enhanced permeability and retention (EPR) effect leads to substantial nonspecific probe accumulation within tumors, limiting our ability to distinguish between probes internalized by cancer cells and those passively retained in the TME^42^. As a result, intensity-based imaging metrics often yield inaccurate estimates of biomarker expression and lack the robustness needed for reliable comparisons across subjects or timepoints.

We recently demonstrated that fluorescence lifetime (FLT) imaging, which measures the decay times of fluorescent probes following excitation by a short laser pulse, provides superior accuracy for tumor delineation even in the presence of high background probe uptake^43–45^. Unlike fluorescence intensity, which is a system-dependent quantity and is susceptible to tissue absorption and scattering, FLT is inherently robust to measurement conditions and tissue optical properties but is sensitive to local tissue microenvironment properties such as pH, polarity and molecular binding^46–48^. Prior work from our group and others has demonstrated that FLT of NIR probes increases following tumor uptake, improving tumor contrast and enabling assessment of treatment response^42,44,49,50^. Notably, tumor-targeted NIR probes internalized by tumor cells exhibit significantly longer FLTs than those nonspecifically retained within the TME^43,45^. Capitalizing on this FLT contrast, our earlier work employed a PD-L1–targeted antibody conjugated to IRDye 800CW (αPDL1-800) in murine orthotopic tumor models and showed that the decay parameter, amplitude, exhibits significantly stronger correlation with *ex vivo* PD-L1 expression than conventional intensity-based measurements^51^.

Here, we demonstrate that tomographic FLT imaging using an asymptotic time-domain (ATD) approach enables reconstruction of the *in vivo* three-dimensional (3D) distribution of αPDL1-800 in deep-seated HCC tumors in mice. This represents an important advancement in molecular imaging for HCC, where accurate biomarker quantification is particularly challenging due to high nonspecific accumulation of antibody-based probes in the liver^52^. *In situ* imaging reveals a significantly longer FLT component $\left(\tau_{L}\right)$ for αPDL1-800 within HCC tumors compared to the shorter FLT component $\left(\tau_{S}\right)$ associated with nonspecifically accumulated αPDL1-800 in the surrounding normal liver. Using this FLT contrast, we define quantitative metrics based on the relative amplitudes of $\tau_{L}$ and $\tau_{S}$ to estimate PD-L1 expression *in vivo*. We further show that a crosstalk between $\tau_{L}$ and $\tau_{S}$ introduces systematic errors in amplitude estimation. To address this, we derive and evaluate four amplitude-based metrics for PD-L1 quantification and demonstrate that a model accounting for the non-zero crosstalk yields the strongest correlation with *ex vivo* PD-L1 expression.

## Materials and Methods

### Cells and culture condition

Cell line authentication and mycoplasma contamination testing were performed prior to all experiments. Cells were used for up to 30 passages after thawing from frozen stocks. The murine HCC cell line, RIL-175 (a p53/Hras mutant line syngeneic to C57Bl/6 mouse strain background), was kindly provided by Dr. Tim Greten (NIH). RIL-175 cells were maintained in Dulbecco’s modified medium (DMEM) with 20% FBS and 1% penicillin/streptomycin. All cells were cultured at 37°C in a humidified incubator with 5% CO_2_ and cells were harvested at 80% confluency for tumor induction.

### Antibody conjugation

A monoclonal anti-PD-L1 antibody (Clone 29E.2A3, Cat# BE0285) was purchased from BioXcell (West Lebanon, NH) and conjugated to IRDye 800CW (cat# 928-38040, Li-COR) according to the manufacturer’s protocol using NHS ester chemistry. Briefly, the antibody was first diluted to 1 mg/mL concentration with PBS (pH 7.4), and then the pH of the protein solution was raised to 8.5 by adding 1 M potassium phosphate buffer. The antibody solution (1 mL) was mixed with IRDye 800CW (60μg) and incubated for 2 hr at room temperature in the dark. The antibody-dye conjugate (αPDL1-800) was purified using a Pierce Zeba desalting spin column (Cat# 89891, Thermo Fisher Scientific, MA). The antibody-to-dye conjugation ratio was determined to be ~2 by measuring UV-Vis absorption.

### Animal models

**Figure 1:**
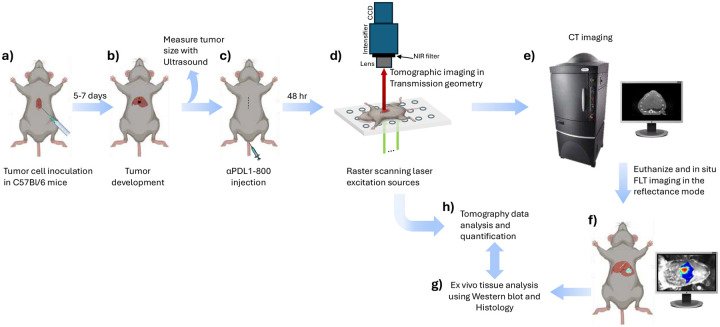
Schematic of orthotopic HCC tumor imaging studies. **(a)** Eight-week-old male C57Bl/6 mice (n = 10) were used for orthotopic implantation of RIL-175 cells into the left lobe of the liver. **(b)** 5–7 days were allowed to establish HCC tumors. Tumor growth was monitored by high-frequency ultrasonography every three days until tumors reached 5–7 mm in diameter. **(c)** Mice bearing orthotopic HCC tumors were intravenously administered αPDL1-800 (150μl,1mg/ml). **(d)** After 48 hours, dorsal and ventral surfaces were shaved, and mice were positioned on a NIR transparent glass-bottom imaging stage with the ventral surface facing the camera. The schematic shows representative positions of the excitation sources (green arrows) below the imaging stage and the fluorescence emission collected by an image intensifier coupled to a CCD camera in the transmission geometry (red arrow). **(e)** Following optical tomography, mice were transferred to an IVIS Spectrum CT system for whole-body CT imaging. **(f)** After *in vivo* imaging, mice were sacrificed, and tumors were imaged *in situ* in the reflectance geometry. *Ex vivo* tumors and normal liver were immediately frozen in liquid nitrogen for subsequent Western blot analysis of PD-L1 expression. **(g)** *Ex vivo* HCC tumors and normal liver were analyzed for PD-L1 expression using Western blots. **(h)** PD-L1 expression metrics determined from *in situ* planar imaging and *in vivo* tomographic imaging were statistically compared with Western blot results. Figure created in part with BioRender.

All animal studies were approved by the Institutional Animal Care and Use Committee (Approval# 2012N000054) in accordance with the animal welfare guidelines at the Massachusetts General Hospital (MGH). Eight weeks old male C57Bl/6 mice (n = 10, wild-type, Jackson Labs) were used for the studies as the RIL-175 cell line is syngeneic to this mouse strain background. Animals were quarantined for 1 week and kept on a normal diet with 12-hour light and dark cycle before the cell implantation. Orthotopic HCC mouse model was established as described in the experiment schematic in Figure 1a, b. RIL-175 cells (1 × 10^6^ cells in 1:1 Matrigel and DMEM supplemented with 20% FBS) were implanted into the left lobe of the liver and tumor growth was monitored using high-frequency ultrasonography every three days. All tumors were allowed to grow until they reached 5–7 mm in diameter (typically within 5–7 days after implantation) in the longest dimension and then were used for *in vivo* TD fluorescence imaging.

### Wide-field TD imaging

After tumor growth, the HCC tumor bearing mice were *IV* administered with αPDL1-800(150μl,1mg/ml) (Fig. 1c). Figure 1d–f shows a schematic of the imaging and data analysis pipeline. 48 hr after αPDL1-800 administration, mice were shaved by applying a thin layer of Nair on both dorsal and ventral surfaces. Mice were then placed on a glass bottom imaging stage with the ventral surface facing the camera (Supplementary Figure 1). Three CT active fiducial markers were placed within the image field of view. Wide-field TD imaging was performed in the transmission mode (Fig. 1d) and a color photograph of the ventral surface of the mouse was acquired. After *in vivo* TD imaging, the imaging stage was transferred into an IVIS Spectrum CT system (PerkinElmer, MA) and whole-body CT imaging was performed (Fig. 1e). Mice were then sacrificed, and tumors were imaged *in situ* (Fig. 1f). The *ex vivo* tumors and normal liver were immediately frozen in liquid nitrogen and subsequently processed for Western blotting to quantify PD-L1 expression (Fig. 1g, h).

#### TD imaging system:

TD imaging was performed using a previously described small-animal fluorescence imaging platform capable of both reflection and transmission geometries^51,53^. The system employed a supercontinuum laser with a tunable filter (EXR-20, SuperK Varia, NKT Photonics; 80 MHz repetition rate, 400–850 nm tuning range) to provide 770 ± 20 nm excitation. Fluorescence emission was detected through an 835 ± 35 nm band-pass filter (AVR Optics) and recorded with a gated intensified CCD camera (LaVision Picostar; 500 V gain; 0.1–1 s integration time; 256 × 344 pixels after 4 × 4 hardware binning). Time-resolved fluorescence images were acquired using a 500 ps gate width with 200 ps step increments, covering a total duration of ~6 ns within each 12.5 ns laser duty cycle.

#### *In vivo* data acquisition in the transmission geometry:

A black cloth with a window matching the dimensions of the mouse torso was placed over the animal to minimize light piping and prevent excitation light leakage from the lateral surfaces. Transmission imaging was performed by scanning the dorsal surface of the mouse with the output of the supercontinuum laser (Fig. 1d). The laser output was coupled into a multimode optical fiber and focused onto the dorsal surface to produce an illumination spot of <1 mm diameter at 8–10 mW power. The illumination beam was sequentially translated across the dorsal surface using a motorized translation stage, with positions spaced 5 mm apart to cover the entire torso. In total, 24 source positions were used to collect TD fluorescence data across 30 time delays in the transmission geometry. At each source position, the system impulse response function was recorded to establish source locations, as described previously^51^.

#### *In situ* data acquisition in reflection geometry:

The output of the supercontinuum laser was coupled to a second multimode optical fiber (Thorlabs, NJ), which delivered the excitation light to a digital micromirror device (DMD) positioned in the reflectance geometry. The DMD expanded the fiber output to uniformly illuminate the ventral surface of the mouse, with approximately 3 mW of power distributed over the illumination area (approximately 6 × 8 cm). Following *in vivo* TD imaging and CT imaging, mice were removed from the imaging stage, euthanized, and dissected to expose the HCC tumor and surrounding normal liver tissue. Care was taken to minimize movement of the internal organs during dissection to preserve the relative anatomical position of the tumor. The mouse carcass was then repositioned on the imaging stage, and in-situ TD fluorescence data were acquired in the reflectance geometry (Fig. 1f). Color photographs were captured to document the location of the HCC tumor within the liver.

### Protein extraction and Western blotting

The HCC tumors and normal liver tissue that were frozen in liquid nitrogen, were first lysed in RIPA buffer. The lysates were then loaded on 8% SDS-polyacrylamide gel at equal amounts of protein (15μg) per well and transferred onto PVDF membranes. The membranes were blocked using 5% non-fat milk in TBST for 1 hr at room temperature. Then, they were probed with a primary antibody against PD-L1 (1:1000, Abcam, ab213480) overnight followed by incubation with an anti-rabbit HRP secondary antibody (1:3000, Cell signaling, 7074S) for 1hr at room temperature. Glyceraldehyde-3-phosphate dehydrogenase (GAPDH) was used as a loading control (1:5000, Cell Signaling, 2118S). Protein bands were detected by Clarity Western ECL Substrate (Bio-Rad, CA) according to the manufacturer’s instructions and developed by autoradiography film (Lab Scientific bioKEMIX, Inc., cat# XAR ALF 2025).

### Data analysis and quantification:

#### Quantification of WB data:

ImageJ was used to quantify the WB images. We first thresholded the WB images to determine the size of each band in pixel units. The total intensity of each PD-L1 band was then calculated. This process was repeated for the corresponding β -actin bands. PD-L1 band intensities were normalized by dividing with the corresponding β -actin band intensities.

#### Tumor volume:

Tumor growth was monitored using high-frequency ultrasound imaging. Animals were imaged in a supine position, and tumors were identified based on anatomical contrast in B-mode images. For each tumor, two orthogonal dimensions were measured from the ultrasound images: the shortest diameter (short axis) and the longest diameter (long axis). Tumor volume was estimated assuming ellipsoidal geometry using the following standard approximation:

(1) $$v=1/2(\text{short}\;\text{axis}{)}^{2}(\text{long}\;\text{axis}),$$

#### *In situ* reflectance data analysis:

We first analyzed the *in situ* TD data acquired in the reflectance geometry to estimate the FLTs of αPDL1-800 within HCC and surrounding normal liver. A color photograph of the *in situ* tumor was first co-registered with the corresponding TD fluorescence image. A region of interest (ROI) encompassing the entire liver was delineated on the color photograph. The liver ROI was then mapped onto the co-registered TD fluorescence image. Within this ROI, the TD fluorescence decays were fitted on a pixel-by-pixel basis using a mono-exponential decay model:

(2) I(t)=a0+a1e−tτ,

where τ represents the FLT and $a_{1}$ corresponds to the amplitude of αPDL1-800 at each pixel. A two-dimensional (2D) fluorescence intensity map of the liver ROI was generated by summing the signal across all time gates. Pixels with fluorescence intensity below 20% of the maximum value were excluded from the mono-exponential fitting.

**Figure 2:**
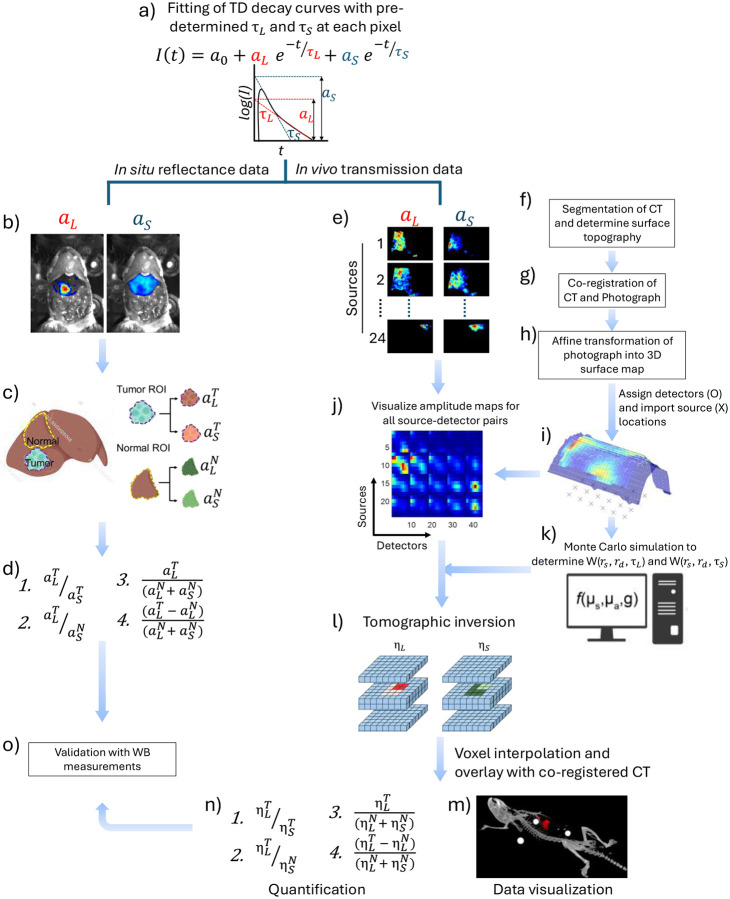
Workflow for *in situ* reflectance and *in vivo* transmission TD data analysis. **(a)** Schematic of a TD fluorescence decay acquired. A linear bi-exponential fit to the asymptotic portion of the decay (consisting of late arriving photons), using pre-determined long ($\tau_{L}$, red) and short ($\tau_{S}$, blue) FLTs of αPDL1-800, was performed to estimate the corresponding decay amplitudes of the long ($a_{L}$, red) and short ($a_{S}$, blue) FLT components. **(b)** Two-dimensional maps of $a_{L}$ and $a_{S}$ from *in situ* liver imaging were co-registered with a corresponding *in situ* photograph of the mouse abdomen. **(c)** Segmentation of amplitude maps into tumor and normal liver regions of interest (ROIs), yielding $a_{L}^{T},a_{S}^{T},a_{L}^{N}$, and $a_{S}^{N}$ for subsequent quantification. **(d)** Four amplitude-based metrics were derived from tumor and normal liver amplitudes to quantify PD-L1 expression while accounting for nonspecific probe uptake and the crosstalk between the two FLT components $\left(\tau_{L},\tau_{S}\right)$. **(e)** *In vivo* tomography data acquired in the transmission geometry were analyzed using a linear bi-exponential fit similar to the reflectance data to generate the amplitude maps, $a_{L}$ and $a_{S}$ for all 24 source positions. **(f–h)** CT-based segmentation of the mouse torso and co-registration with white-light photographs using CT fiducials, followed by affine transformation to generate a surface topography and volumetric mouse geometry. **(i)** Assignment of source locations (‘x’) and 32 detectors (‘o’) which are pixels on the CCD image, arranged in a 4×8 grid in the transmission geometry. **(j)** Visualization of amplitude maps for all source-detector pairs (24 × 32) to assess data quality. Measurement pairs corrupted by excitation light leakage are excluded from further analysis. **(k)** Monte Carlo–based forward modeling using the CT-derived mouse volume and mapped source–detector positions to compute fluorescence sensitivity matrices. **(l)** Tomographic inversion to reconstruct three-dimensional fluorescence yield distributions of tumor $\left(\eta_{L}\right)$ and normal $\left(\eta_{S}\right)$ probe fractions. **(m)** Interpolation of reconstructed 3D yield distributions, $\eta_{L}$ and $\eta_{S}$ to the CT voxels grid and visualization as overlays with CT images for anatomical localization within the liver. **(n)** Derivation of four fluorescence yield–based metrics from tumor and normal liver volumes. **(o)** Comparison of amplitude-based and yield-based metrics with PD-L1 expression measured by Western blot to identify the optimal quantitative parameter. Figure created in part with BioRender.

Subsequently, two additional ROIs were defined to isolate the tumor region and the surrounding normal liver based on the color photograph. Pixel-wise FLTs within each ROI were extracted and tabulated. Based on prior work demonstrating that αPDL1-800 exhibits significantly longer FLTs in PD-L1 expressing tumor regions compared to PD-L1 negative regions^51^, the mean of the highest 10% of FLT values within the tumor ROI was used to represent the long FLT component $\left(\tau_{L}\right)$, while the mean of the lowest 10% of FLT values within the normal liver ROI was used to represent the short FLT component $\left(\tau_{S}\right)$. The $\tau_{L}$ and $\tau_{S}$ estimated in this manner provided approximations of the PD-L1 associated and free αPDL1-800 fractions *in vivo* and were subsequently used in amplitude analysis. Using the $\tau_{L}$ and $\tau_{S}$ values, a bi-exponential fit was performed on the TD fluorescence decay (Fig. 2a) in the full liver ROI, according to the model:

(3) I(t)=a0+aL⋅e−tτL+aS⋅e−tτS

Where $a_{L}$ and $a_{S}$ represent the amplitudes of the long and short FLT fractions of αPDL1-800, respectively. 2D maps of $a_{L}$ and $a_{S}$ were overlaid onto the *in situ* photographs of the HCC tumor (Fig. 2b). In our prior work, we employed the above-mentioned bi-exponential analysis of TD fluorescence data, which enabled quantitative assessment of PD-L1 expression in a superficial orthotopic breast cancer model^51^.

#### Quantification of *in situ* reflectance data:

The tumor and normal liver ROIs were first applied to segment the amplitude maps, separating $a_{L}$ into tumor $\left(a_{L}^{T}\right)$ and normal liver $\left(a_{L}^{N}\right)$ components, and $a_{S}$ into tumor $\left(a_{S}^{T}\right)$ and normal liver $\left(a_{S}^{N}\right)$ components (Fig. 2c). The resulting four amplitude maps were averaged across all pixels, with the mean $a_{L}^{T}$ representing the long-FLT fraction of αPDL1-800 within the tumor. To account for background probe uptake in the normal liver, free αPDL1-800 in the TME, and errors from crosstalk between $\tau_{L}$ and $\tau_{S}$, four additional metrics reflecting PD-L1 expression were computed (Fig. 2d):

$$\text{Metric}\;1=\frac{a_{L}^{T}}{a_{S}^{T}},$$

$$\text{Metric}\;2=\frac{a_{L}^{T}}{a_{S}^{N}},$$

$$\text{Metric}\;3=\frac{a_{L}^{T}}{\left(a_{L}^{N}+a_{S}^{N}\right)},$$

$$\text{Metric}\;4=\frac{\left(a_{L}^{T}-a_{L}^{N}\right)}{\left(a_{L}^{N}+a_{S}^{N}\right)}.$$

These four metrics together were compared using scatter plots against PD-L1 expression measured by WB analysis. Tumor-to-background intensity ratio (TBR) was computed as the ratio of the mean tumor intensity to the mean fluorescence intensity of the normal liver ROI.

#### *In vivo* transmission data analysis:

Tomographic reconstruction of *in vivo* TD fluorescence data was performed using the Asymptotic Time Domain (ATD) method as previously described^54^. ATD tomographic reconstruction generated three-dimensional (3D) fluorescence yield distributions (η) of αPDL1-800 in HCC tumors and adjacent normal liver tissue. For each source position, TD fluorescence data were screened to remove saturated pixels. Source locations near the edge of the mouse torso often presented >20% saturated pixels due to excitation light leakage. In such cases, all decay data corresponding to that source were excluded from further analysis. The TD fluorescence decays for each source were then analyzed using the ATD approach. In the asymptotic regime, the measured fluorescence signal, $U_{\left(x,m\right)}$, is expressed as a sum of exponentials:

(4) U(x,m)rs,rd,t→aLrs,rde−tτL+aSrs,rde−tτS,

where $r_{s}$ and $r_{d}$ denote the source and detector positions, respectively. The decay portion of the TD data was fit to equation (4) using predetermined $\tau_{L}$ and $\tau_{S}$, thereby separating their corresponding amplitudes ($a_{L}$ and $a_{S}$). Fitting was performed with an unconstrained nonlinear least squares minimization method. The $a_{L}$ and $a_{S}$ maps at each source locations reflect the spatial distributions of the long and short FLT populations of αPDL1-800, respectively (Fig. 2e).

The anatomical geometry of the mouse was derived by co-registering CT data with a photograph of the mouse using three CT-active fiducial markers present in both datasets, providing fixed reference points for accurate spatial alignment. The CT volume was then down-sampled to a voxel size of 1 mm^3^. Using the CT geometry as a reference, the contour of the mouse surface was extracted and used to affine transform the photograph, generating a 3D surface topography and a digitized volume of the mouse torso (Fig. 2f–h).

On the CCD camera image, 32 detector positions were arranged in a 4 × 8 grid (separated by 5 pixels), resulting in 768 source-detector pairs. The source (obtained from IRF measurements) and detector coordinates, $r_{s}$ and $r_{d}$ respectively, were then mapped onto the mouse surface topography (Fig. 2i). The amplitudes $a_{L}$ and $a_{S}$ for each source-detector pair were visualized as a 2D map as a quality control measure to ensure that data from all relevant source-detector measurements were included in the downstream analysis (Fig. 2j). These source-detector coordinates formed the input for forward modeling of photon propagation through the 3D mouse volume (Fig. 2k). The forward model was computed using Monte Carlo (MC) photon transport simulations implemented in MCX on a GPU platform. For each source-detector pair, 10^7^ photons were propagated through the digitized mouse volume. To ensure continuity of the segmented volume, a morphological closing operation was applied. Homogeneous optical properties were assumed throughout the tissue, with an absorption coefficient $\left(\mu_{a}\right)$ of 0.1 cm^−1^ and a reduced scattering coefficient $\left(\mu_{s}^{\prime }\right)$ of 10 cm^−1^. Using these parameters, fluorescence sensitivity matrices (Green’s function), $W\left(r_{s},r_{d},r^{\prime }\right)$, for the long $\left(\tau_{L}\right)$ and short $\left(\tau_{S}\right)$ FLTs were computed to describe photon propagation within the medium at both excitation and emission wavelengths. The amplitudes $a_{L}$ and $a_{S}$ for each source-detector pair are related to the fluorescence sensitivity matrix, $W\left(r_{s},r_{d},r^{\prime }\right)$ and the yield distributions of the long and short FLT components, $\eta_{L}\left(r^{\prime }\right)$ and $\eta_{S}\left(r^{\prime }\right)$ respectively, as follows:

(5) $$a_{L}\left(r_{s},r_{d}\right)=\int W\left(r_{s},r_{d},r^{\prime }\right)\cdot \eta_{L}\left(r^{\prime }\right)dr^{\prime },$$

The yield distributions $\eta_{L}\left(r^{\prime }\right)$ and $\eta_{S}\left(r^{\prime }\right)$ were obtained by inverting the sensitivity matrix using a Tikhonov-regularized least-squares minimization algorithm. For clarity, $W\left(r_{s},r_{d},r^{\prime }\right)$ is denoted simply as W, and the inverse solution is written as:

(6) $$\eta_{L}\left(r^{\prime }\right)=W^{-1}a_{L}\left(r_{s},r_{d}\right),$$

with

(7) $$W^{†}=W^{T}{\left(WW^{T}+\lambda \alpha I\right)}^{-1},$$

where $\alpha =\text{max}\left(\text{diag}\left(WW^{T}\right)\right)$ and λ is the regularization parameter. The ATD tomographic reconstruction using equation (6) provided 3D distributions of $\eta_{L}$ and $\eta_{S}$ in each voxel $\left(r^{\prime }\right)$ within the mouse torso (Fig. 2l). Reconstructions for each animal were performed using ten different λ values. The λ that produced $\eta_{L}$ distributions localized consistently within the liver across all animals was selected for the final analysis. The liver region was segmented from the CT images using Living Image software (PerkinElmer). The reconstructed $\eta_{L}$ and $\eta_{S}$ distributions were interpolated in MATLAB to match the voxel grid of the CT data (Fig. 2m). This allowed the fluorescence yield maps to be co-registered with the CT anatomy and overlaid in ImageJ (NIH, Version 1.54f) for anatomical context. Voxels in the upper and lower boundary planes of the 3D reconstruction were excluded from further analysis to minimize potential boundary-related artifacts from the source and detector positions. This exclusion is a standard practice in diffuse optical tomography (DOT) to improve the accuracy of the reconstructed distributions.

#### Quantification of *in vivo* transmission data:

The interpolated $\eta_{L}$ and $\eta_{S}$ distributions were first thresholded at 80%, excluding the lowest 20% of voxels from further quantification to reduce noise. To define tumor boundaries, the *in situ* tumor photograph was visually compared with the CT - $\eta_{L}$ overlay, and a tumor ROI was delineated on the first plane of the $\eta_{L}$ distribution corresponding to the ventral surface of the mouse torso. A tumor volume was then generated by extending this ROI through the remaining planes of the $\eta_{L}$ distribution. Similarly, a normal liver ROI was delineated on the first plane of the $\eta_{S}$ distribution and extended through the remaining planes to create a volumetric normal liver ROI. These volumetric ROIs were subsequently applied to segment the fluorescence yield distributions separating $\eta_{L}$ into tumor $\left(\eta_{L}^{T}\right)$ and normal liver $\left(\eta_{L}^{N}\right)$ components, and $\eta_{S}$ into tumor $\left(\eta_{S}^{T}\right)$ and normal liver $\left(\eta_{S}^{N}\right)$ components. Voxels with no yield values were excluded, and the mean values of the remaining voxels within $\eta_{L}^{T},\eta_{L}^{N},\eta_{S}^{T}$, and $\eta_{S}^{N}$ were calculated. the mean $\eta_{L}^{T}$ was used to represent the PD-L1 bound fraction of αPDL1-800 within the tumor. Consistent with the *in situ* data analysis, four additional metrics reflecting PD-L1 expression were computed (Fig. 2n):

$$\text{Metric}\;1=\frac{\eta_{L}^{T}}{\eta_{S}^{T}},$$

$$\text{Metric}\;2=\frac{\eta_{L}^{T}}{\eta_{S}^{N}},$$

$$\text{Metric}3=\frac{\eta_{L}^{T}}{\left(\eta_{L}^{N}+\eta_{S}^{N}\right)},$$

$$\text{Metric}\;4=\frac{\left(\eta_{L}^{T}-\eta_{L}^{N}\right)}{\left(\eta_{L}^{N}+\eta_{S}^{N}\right)}.$$

These four parameters together with $\eta_{L}^{T}$, were compared with PD-L1 expression levels determined by WB analysis (Fig. 2o).

### Statistics:

FLT-based metrics of PD-L1 expression, including amplitude and yield distributions, were plotted for each mouse against PD-L1 levels measured by Western blot. Pearson’s correlation coefficient $\left(r^{2}\right)$ was calculated to assess associations between the measures. Statistical significance was determined using a two-tailed Mann–Whitney U test, with P values < 0.05 considered significant: *, P<0.05;**,P<0.01.

## Results

### Heterogeneous PD-L1 expression across orthotopic HCC tumors

**Figure 3:**
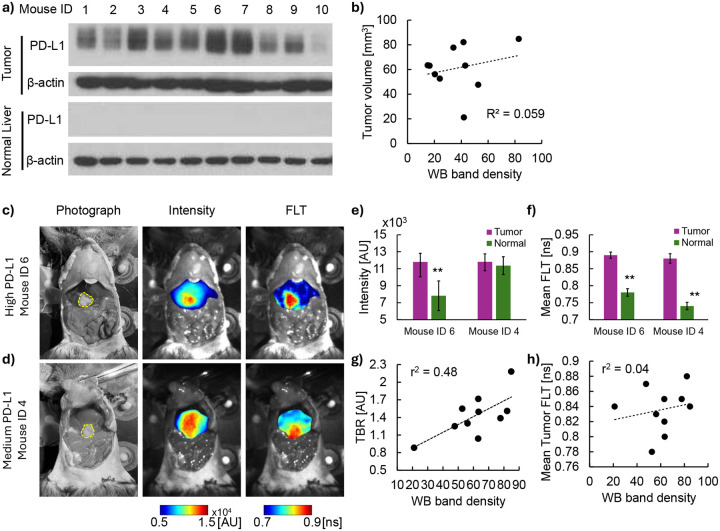
*In situ* FLT imaging and PD-L1 characterization of orthotopic HCC tumors. **(a)** Western blot analysis of PD-L1 expression in HCC tumors (top) and matched normal liver (bottom) from ten mice. β -actin was used as a loading control. Tumors showed substantial inter-tumoral variability in PD-L1 expression, while all normal liver samples showed no detectable PD-L1. Mouse IDs 1–10 are shown above the plot. **(b)** Correlation between tumor volume and PD-L1 band density from Western blots. PD-L1 expression did not correlate with tumor volume (r^2^ = 0.059). **(c–d)** *In situ* fluorescence images of two representative tumors with **(c)** high PD-L1 expression (Mouse ID 6) and **(d)** medium PD-L1 expression (Mouse ID 4). Grayscale photographs (left) of the exposed liver region are shown alongside pseudo-colored fluorescence intensity (middle) and FLT (right) maps. Tumors are outlined with yellow dashed lines. **(e)** Mean fluorescence intensity in tumor (magenta) and normal liver (green) for Mouse 6 and Mouse 4. Mouse 6 showed significantly higher tumor intensity compared to the normal liver, whereas Mouse ID 4 showed comparable intensities between tumor and normal liver. **(f)** Mean fluorescence lifetimes in tumor (magenta) and normal liver (green) for Mouse 6 and Mouse 4. Both animals demonstrated significantly increased tumor FLTs compared to liver. **(g–h)** Correlation of **(g)** tumor-to-background intensity ratio (TBR) and **(h)** mean tumor FLT with PD-L1 expression across all ten mice. TBR showed moderate correlation with PD-L1 expression (r^2^ = 0.48), whereas mean FLT exhibited poor correlation (r^2^ = 0.04). p-value **<0.01.

To characterize the degree of heterogeneity in the RIL-175 orthotopic HCC model, we first assessed PD-L1 expression in tumors collected from ten C57BL/6 mice implanted and harvested under identical experimental conditions. Western blot analysis demonstrated a broad range of PD-L1 expression across tumors (Figure 3a, top), despite the tumors being initiated on the same day in genetically identical hosts. Adjacent normal liver from each animal showed no detectable PD-L1 signal (Figure 3a, bottom). Although the tumor inoculation protocol was standardized, tumor volumes varied widely at the time of imaging and tissue collection. To evaluate whether PD-L1 expression heterogeneity was driven by tumor size, we compared Western blot band intensities to tumor volume across all ten animals. The resulting correlation was negligible (Figure 3b, r^2^ = 0.059), indicating that PD-L1 variability arises from intrinsic biological heterogeneity rather than differences in tumor burden.

To test whether FLT imaging reflected this PD-L1 expression heterogeneity, we analyzed *in situ* fluorescence intensity and FLT maps from two representative mice: one with high PD-L1 expression (Mouse 6) and one with medium PD-L1 expression (Mouse 4). In both cases, grayscale photographs of the exposed liver region were overlaid with pseudocolor intensity and FLT maps, and tumors were delineated manually (Figures 3c–3d). For Mouse 6, both fluorescence intensity and FLT were visibly elevated in the tumor relative to normal liver. In contrast, Mouse 4 exhibited comparable tumor and liver fluorescence intensities, but a persistently longer tumor FLT. Quantitative analysis of tumor and normal liver ROIs revealed Mouse 6 exhibited a significantly higher tumor intensity than liver, while Mouse 4 showed comparable intensities (Figure 3e). However, both mice demonstrated significantly prolonged tumor FLTs relative to normal liver (Figure 3f; p < 0.05). These results illustrate that FLT provides superior tumor contrast than intensity in cases of modest PD-L1 expression, although mean FLT values remained similar between the two tumors.

To determine whether either intensity or FLT reflects PD-L1 expression across the full cohort, we correlated tumor-to-background intensity ratio (TBR) and mean tumor FLT with PD-L1 expression measured via WB. TBR exhibited a moderate correlation (Fig. 3g; r^2^ = 0.48), whereas mean FLT showed minimal correlation (Fig. 3h; r^2^ = 0.04). The relatively improved performance of TBR over mean FLT likely reflects its sensitivity to total probe accumulation, which is partially driven by PD-L1 expression. In contrast, mean FLT is largely independent of probe concentration and therefore does not capture variability in αPDL1-800 uptake across tumors. However, the correlation between TBR and PD-L1 expression remains limited because intensity measurements cannot distinguish between PD-L1 mediated tumor uptake and nonspecifically retained αPDL1-800 within the TME and in the surrounding normal liver. Overall, Figure 3 reveals substantial PD-L1 heterogeneity across HCC tumors and demonstrates that TBR and mean FLT do not reliably reflect PD-L1 expression, particularly in the liver where nonspecific probe uptake is high. TBR and mean FLT values for individual tumors are provided in supplementary Tables 1 and 2, respectively.

### Bi-exponential analysis of TD fluorescence data and amplitude-based metrics for PD-L1 quantification

**Figure 4:**
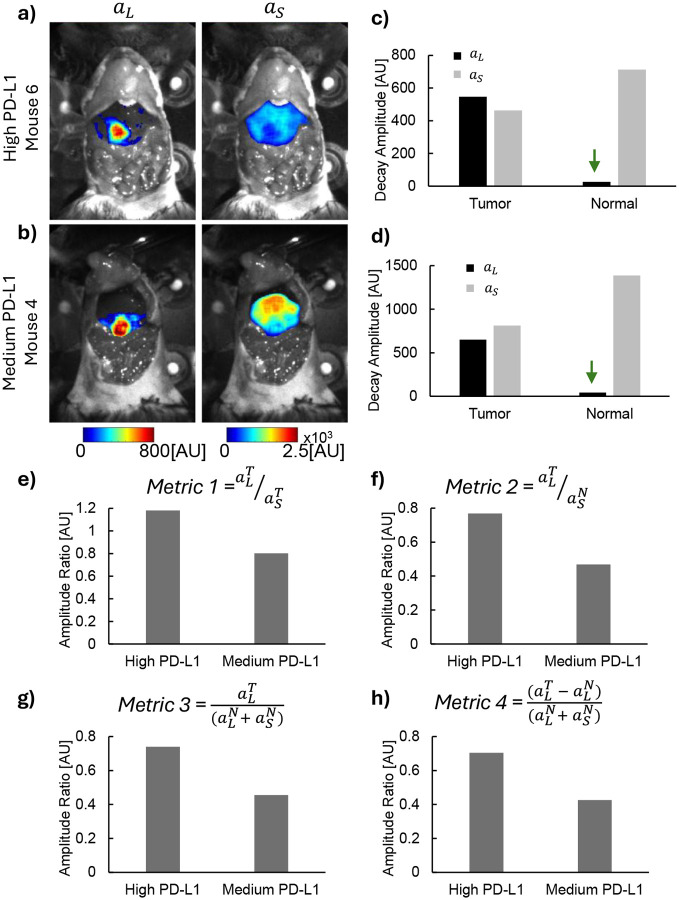
Bi-exponential amplitude analysis of *in situ* TD data. Representative amplitude maps of the long ($a_{L}$, left) and short ($a_{S}$, right) FLT fraction for tumors with **(a)** high PD-L1 expression (Mouse #6) and **(b)** medium PD-L1 expression (Mouse #4). The $a_{L}$ maps delineate the tumor, whereas the $a_{S}$ maps show nonspecific αPDL1-800 uptake in both tumor and surrounding normal liver. **(c–d)** Quantification of $a_{L}$ (black) and $a_{S}$ (gray) in tumor and normal liver ROIs for Mouse #6 **(a)** and Mouse #4 **(b)**. Mouse 6 showed higher tumor $a_{L}$ relative to $a_{S}$, whereas mouse 4 showed lower tumor $a_{L}$ compared to $a_{S}$. Both mice showed higher tumor $a_{L}$ compared to the $a_{L}$ in the normal liver. However, a small but measurable $a_{L}$ in the normal liver (green arrows in **c** and **d**) was present, indicating crosstalk between $\tau_{L}$ and $\tau_{S}$ from the linear bi-exponential fit. **(e–h)** Evaluation of four amplitude-based metrics derived from tumor $\left(a_{L}^{T},a_{S}^{T}\right)$ and normal liver $\left(a_{L}^{N},a_{S}^{N}\right)$ amplitudes. **(e)** Metric 1, defined as $a_{L}^{T}/a_{S}^{T}$, **(f)** Metric 2, defined as $a_{L}^{T}/a_{S}^{N}$, and **(g)** metric 3, defined as $a_{L}^{T}/\left(a_{L}^{N}+a_{S}^{N}\right)$. **(h)** metric 4, defined as $\left(a_{L}^{T}-a_{L}^{N}\right)/\left(a_{L}^{N}+a_{S}^{N}\right)$, which subtracts the crosstalk component in normal tissue, $a_{L}^{N}$ from $a_{L}^{T}$ in the tumor. All four normalized metrics correctly ranked the PD-L1 expression of Mouse #6 above that for Mouse #4, which had a lower PD-L1 expressing tumor.

Although the mean tumor FLT measured *in situ* did not correlate with *ex vivo* measures of αPD−L1 expression, tumors consistently exhibited longer FLT values for αPDL1-800 compared to the surrounding normal liver (Fig. 3d, Supplementary Table 2). This FLT contrast suggests the presence of a distinct long-FLT component within tumors that is associated with PD-L1 expression, whereas the normal liver signal is dominated by a shorter-FLT component. We capitalized on this FLT separation to derive quantitative metrics of PD-L1 expression. As described in the Methods section, the long FLT component $\left(\tau_{L}\right)$ was defined as the mean of the highest 10% of tumor FLT values, while the short FLT component $\left(\tau_{S}\right)$ was defined as the mean of the lowest 10% of liver FLT values. These predefined $\tau_{L}$ and $\tau_{S}$ values were then applied in a bi-exponential model to decompose the TD fluorescence decay at each pixel, yielding two spatially resolved amplitude maps corresponding to the relative contributions of the long $\left(a_{L}\right)$ and short $\left(a_{S}\right)$ FLT components (Fig. 4a, b).

Representative $a_{L}$ and $a_{S}$ maps from Mouse 6 (high PD-L1) and Mouse 4 (medium PD-L1) are shown in Figures 4a and 4b, respectively. The $a_{L}$ maps (Fig. 4a and 4b, first column) clearly delineated the tumors, whereas the $a_{S}$ maps (Fig. 4a and 4b, second column) showed nonspecific distribution of αPDL1-800 in both tumor and surrounding normal liver. This separation emphasizes that nonspecific αPDL1-800 uptake constitutes a significant portion of the total fluorescence signal, consistent with the limited predictive power of TBR measurements. Further quantification of $a_{L}$ and $a_{S}$ revealed clear differences between tumor and normal liver ROIs. For Mouse 6, tumor ROI was dominated by the long FLT fraction $\left(a_{L}\right)$ and the normal liver ROI was dominated by the short FLT fraction $\left(a_{S}\right)$ (Fig. 4c).

For Mouse 4, both tumor and normal liver ROIs were dominated by the short FLT fraction $\left(a_{S}\right)$ (Fig. 4d). Notably, a substantial amplitude of the long FLT fraction $\left(a_{L}\right)$ was consistently observed in normal liver (Fig. 4c and 4d, green arrows; Supplementary Table 2) despite the absence of PD-L1 expression, reflecting FLT crosstalk between $\tau_{L}$ and $\tau_{S}$ that must be addressed analytically.

To systematically account for nonspecific αPDL1-800 uptake and crosstalk between $\tau_{L}$ and $\tau_{S}$, we developed four amplitude-based metrics using the decay amplitudes from tumor $\left(a_{L}^{T},a_{S}^{T}\right)$ and normal liver $\left(a_{L}^{N},a_{S}^{N}\right)$ ROIs. Metrics 1 and 2 normalize the $a_{L}$ from the tumor ROI $\left(a_{L}^{T}\right)$ by the amplitude of the short FLT components from the tumor $\left(a_{S}^{T}\right)$ and normal liver $\left(a_{S}^{N}\right)$, respectively. In Metric 1, normalization by $a_{S}^{T}$ accounts for nonspecific αPDL1-800 accumulation within the TME, which can vary with tumor-stroma composition and degree of vascular leakage. In Metric 2, normalization by $a_{S}^{N}$ reflects background probe accumulation in normal liver and partially corrects for inter-animal variability in probe distribution. Metric 3, defined as $a_{L}^{T}/\left(a_{L}^{N}+a_{S}^{N}\right)$, normalizes $a_{L}^{T}$ by the total αPDL1-800 amplitude in the normal liver, thereby accounting for both $a_{S}^{N}$ (nonspecific uptake) and $a_{L}^{N}$ the amplitude fraction that was incorrectly assigned to the long FLT $\left(\tau_{L}\right)$ fraction of αPDL1-800 in the normal liver (crosstalk).

Metric 4, defined as $\left(a_{L}^{T}-a_{L}^{N}\right)/\left(a_{L}^{N}+a_{S}^{N}\right)$, further refines this approach by subtracting the long FLT contribution observed in the normal liver ROI $\left(a_{L}^{N}\right)$ prior to normalization. This correction reduces bias arising from $\tau_{L}-\tau_{S}$ crosstalk and provides a more specific estimate of the PD-L1 associated long FLT fraction of αPDL1-800 within the tumor. Although the absolute tumor amplitude (Supplementary Table 2) of the long FLT component $\left(a_{L}^{T}\right)$ was higher in the medium PD-L1 expressing tumor (Mouse 4; $a_{L}^{T}=651\;\text{AU}$) than in the high PD-L1 expressing tumor (Mouse 6; $a_{L}^{T}=547.5\;\text{AU}$), normalization using Metrics 1–4 correctly ranked the high PD-L1 tumor above the medium PD-L1 tumor (Fig. 4e–h). These results highlight the importance of normalization to reduce the impact of nonspecific uptake and $\tau_{L}-\tau_{S}$ crosstalk. Figure 4 further shows that bi-exponential analysis using predefined $\tau_{L}$ and $\tau_{S}$ enables separation of distinct FLT components and provides a basis for evaluating these metrics across the full cohort.

### Amplitude-based metrics correlate with PD-L1 expression across the full mouse cohort

**Figure 5:**
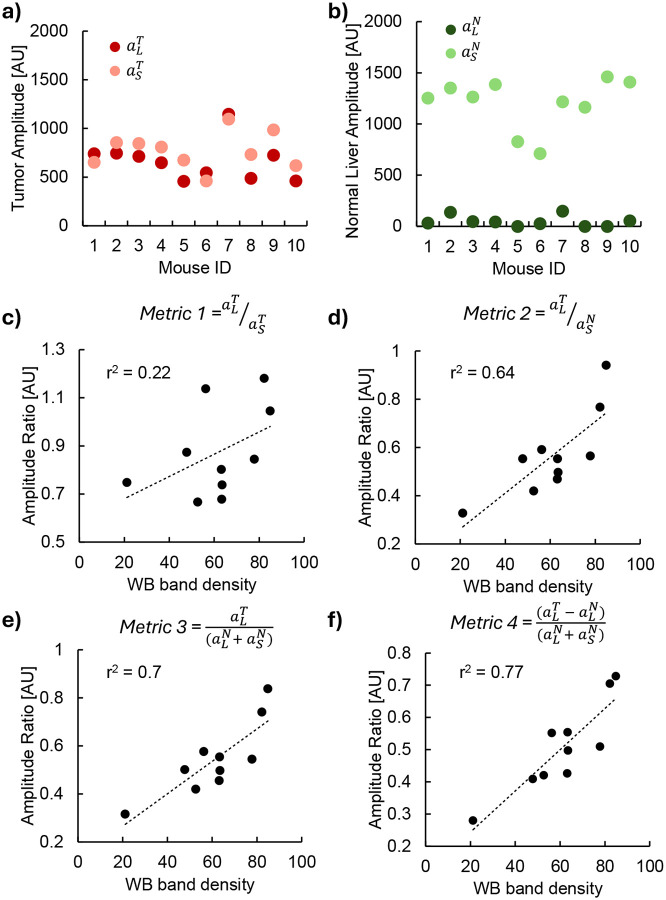
Comparison of four decay amplitude-based metrics for PD-L1 quantification across mice. **(a)** Decay amplitudes for the long FLT $\left(a_{L}^{T}\right)$ (red) and short FLT $\left(a_{S}^{T}\right)$ (pink) within the tumors across all ten mice. In seven tumors (Mouse IDs 2, 3, 4, 5, 8, 9, 10 from Fig. 3a), $a_{S}^{T}$ exceeded $a_{L}^{T}$, indicating that nonspecific αPDL1-800 accumulation (short FLT) dominated the total fluorescence signal. **(b)** The decay amplitudes in the liver, for the long $\left(a_{L}^{N}\right)$ (dark green) and short $\left(a_{S}^{N}\right)$ (light green) FLT components. As expected, the amplitude of the short FLT component, $a_{S}^{N}$, was consistently higher than $a_{L}^{N}$ in the normal liver in all animals. Nevertheless, a measurable $a_{L}^{N}$ was detected in seven mice, reflecting lifetime crosstalk between $\tau_{L}$ and $\tau_{S}$ despite the absence of PD-L1 in normal liver. **(c–f)** Correlation of four amplitude-based metrics with PD-L1 protein levels measured by Western blot. **(c)** Metric 1 $\left(a_{L}^{T}/a_{S}^{T}\right)$ showed a weak correlation with PD-L1 expression (r^2^ = 0.22). **(d)** Metric 2 $\left(a_{L}^{T}/a_{S}^{N}\right)$ and **(e)** Metric 3 $\left(a_{L}^{T}/\left(a_{L}^{N}+a_{S}^{N}\right)\right)$ demonstrated moderately strong correlations (r^2^ = 0.64 and 0.7, respectively). **(f)** Metric 4 $\left(\left(a_{L}^{T}-a_{L}^{N}\right)/\left(a_{L}^{N}+a_{S}^{N}\right)\right)$, which subtracts the amplitude cross-talk between $\tau_{L}$ and $\tau_{S}$ and normalizes by the total normal liver amplitude, showed the strongest correlation with PD-L1 expression (r^2^ = 0.77).

Having established the amplitude-based PD-L1 quantification framework in representative tumors, we next evaluated these metrics across the full cohort of ten tumors. Tumor amplitudes displayed substantial variability, with seven tumors exhibiting higher amplitude of the short FLT $\left(a_{S}^{T}\right)$ than long FLT $\left(a_{L}^{T}\right)$ components (Fig. 5a), indicating that nonspecific signal contributes significantly to the total fluorescence within the tumor. In normal liver (Fig. 5b), the short FLT amplitudes $\left(a_{S}^{N}\right)$ were consistently higher than the long FLT amplitudes $\left(a_{L}^{N}\right)$, as expected. However, a detectable $a_{L}^{N}$ was present in seven livers, consistent with a crosstalk between $\tau_{L}$ and $\tau_{S}$.

We then evaluated the ability of the four amplitude-based metrics to quantify PD-L1 expression across the entire mouse cohort. Metric 1 (Fig. 5c) showed a weak correlation with WB-based PD-L1 levels, with a correlation coefficient (r^2^) of 0.22. In contrast, Metrics 2 (Fig. 5d) and 3 (Fig. 5e) demonstrated substantially stronger correlations, with r^2^ value of 0.64 and 0.7, respectively. Metric 4, which subtracts the long FLT amplitude observed in normal liver ($a_{L}^{N}$; crosstalk) from the long FLT amplitude in the tumor $\left(a_{L}^{T}\right)$ and normalizes by total normal liver amplitude $\left(a_{L}^{N}+a_{S}^{N}\right)$, yielded the strongest correlation with PD-L1 expression (r^2^ = 0.77; Fig. 5f). These results establish Metric 4 as the most robust amplitude-based parameter for PD-L1 quantification.

### ATD-FLT tomography accurately localizes orthotopic HCC tumors and enables *in vivo* quantification of PD-L1 expression

**Figure 6:**
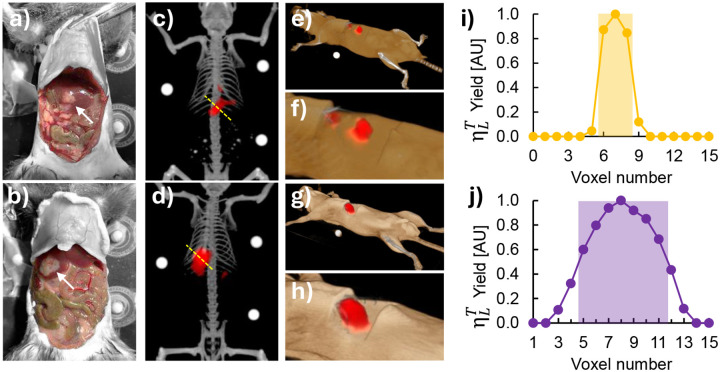
ATD-FLT tomographic reconstruction localizes and resolves orthotopic HCC tumors of different sizes. **(a, b)** *In situ* white-light photographs of mice bearing a small (~3.7 mm; **a**) and a large (~7 mm; **b**) HCC tumor, with tumor locations indicated by white arrows. The abdominal region is shown in RGB, and the remainder of the torso is shown in grayscale. **(c, d)** Reconstructed yield distributions of the long FLT fraction ($\eta_{L}^{T}$, red) for the small **(c)** and large **(d)** tumors, overlaid on the CT-derived mouse skeleton (gray). **(e–h)** Three-dimensional renderings of $\eta_{L}^{T}$ in the context of CT-derived skeleton (gray) and soft tissue (brown) for the small **(e, f)** and large **(g, h)** tumors. In the magnified views **(f, h)**, soft tissue in the abdominal region is partially removed to visualize the $\eta_{L}^{T}$ volumes, which localize to the tumor-bearing portion of the liver. The $\eta_{L}^{T}$ distributions for the large tumor **(d, g, h)** are broader and deeper than those for the small tumor **(c, e, f)**, consistent with tumor size and location identified in the white-light photographs. **(i, j)** Line profiles across the $\eta_{L}^{T}$ distributions along the dashed yellow lines, 15 voxels in length, shown in **(c)** and **(d)**. The full width at half maximum spanned approximately three voxels for the small tumor (yellow shaded region, **i)** and seven voxels for the large tumor (purple shared region, **j**), corresponding to reconstructed tumor diameters of ~3 mm and ~7 mm.

Following validation of the amplitude-based metrics *in situ*, we next extended this framework to three-dimensional imaging using asymptotic time-domain (ATD) FLT tomography. ATD reconstruction was applied to *in vivo* TD transmission data to localize the long- and short-FLT components within orthotopic HCC tumors, and Metric 4 was used for quantitative assessment of PD-L1 expression. Representative tomographic results from two mice bearing a small (~3.7 mm) and a large (~7 mm) HCC tumor are shown in Figure 6. White-light photographs identified tumor locations (Fig. 6a, b; arrows) and served as anatomical references. Reconstructed $\eta_{L}$ and $\eta_{S}$ volume distributions first segmented into tumor $\left(\eta_{L}^{T},\eta_{S}^{T}\right)$ and normal liver $\left(\eta_{L}^{N},\eta_{S}^{N}\right)$ ROIs, and the $\eta_{L}^{T}$ volumes (Fig. 6c, d; red) were overlaid on CT-derived skeletal structures (Fig. 6c, d; gray). For both tumors, $\eta_{L}^{T}$ localized to the liver region and matched tumor positions observed in the photographs (Fig. 6a, b). Three-dimensional renderings incorporating CT-derived anatomy further confirmed accurate spatial localization and depth of the $\eta_{L}^{T}$ distribution (Fig. 6e–h). Soft tissue (Fig. 6e–h, brown) segmented from CT images were removed from the upper abdomen area to enable clear visualization of $\eta_{L}^{T}$ distribution within the tumor bearing region of the liver. Line profile analysis across the reconstructed $\eta_{L}^{T}$ volumes (Fig. 6i, j) showed that the full width at half maximum (FWHM) spanned approximately three voxels for the small tumor and seven voxels for the large tumor. Given a voxel size of 1 mm^3^, these correspond to tumor diameters of ~3 mm and ~7 mm, respectively, demonstrating that reconstructed $\eta_{L}^{T}$ volumes scale appropriately with true tumor size. Together, these results establish that ATD-FLT tomography accurately localizes HCC tumors *in vivo* and provides volumetric information consistent with anatomical ground truth.

**Figure 7:**
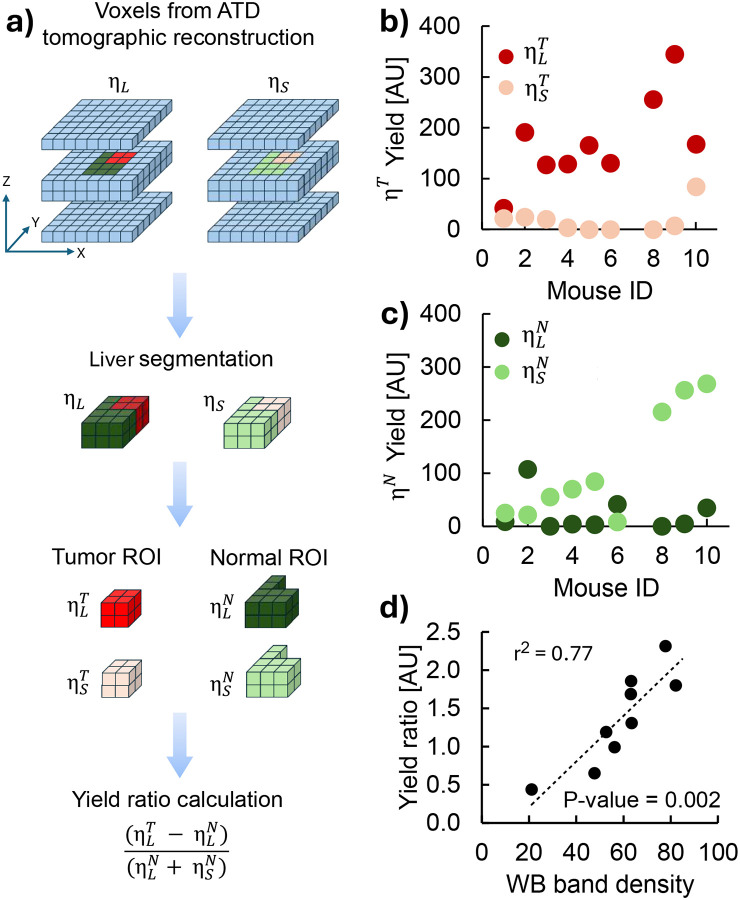
Quantification of αPDL1-800 fluorescence yields and correlation with PD-L1 expression. **(a)** Schematic of the tomographic quantification workflow. ATD-FLT reconstruction generates volumetric maps of fluorescence yields of the long ($\eta_{L}$, left) and short ($\eta_{S}$, right) FLT components. CT-based segmentation identifies liver voxels, from which tumor and normal liver ROIs are defined and used to compute Metric 4 $\left(\left(\eta_{L}^{T}-\right.\left.\eta_{L}^{N}\right)/\left(\eta_{L}^{N}+\eta_{S}^{N}\right)\right)$. **(b)** Tumor yields for the long $\left(\eta_{L}^{T}\right)$ and short $\left(\eta_{S}^{T}\right)$ FLT components across nine mice. In all tumors, $\eta_{L}^{T}$ exceeded $\eta_{S}^{T}$. **(c)** Normal liver yields for the long $\left(\eta_{L}^{N}\right)$ and short $\left(\eta_{S}^{N}\right)$ FLT components. Seven out of nine (Mouse ID 1, 3, 4, 5, 8, 9, 10) mice exhibited substantially higher $\eta_{S}^{N}$ compared to $\eta_{L}^{N}$, consistent with the absence of PD-L1 expression in normal liver. Two mice (Mouse ID 2 and 6) showed higher $\eta_{L}^{N}$ than $\eta_{S}^{N}$, reflecting high $\tau_{L}-\tau_{S}$ crosstalk. **(d)** Correlation between Metric 4 and PD-L1 protein levels measured by Western blot (r^2^ = 0.77, p < 0.01).

We next performed quantitative analysis using the tomographic workflow outlined in Figure 7a. After segmenting liver voxels from CT and co-registering $\eta_{L}$ and $\eta_{S}$ volumes, values for $\eta_{L}^{T},\eta_{S}^{T},\eta_{L}^{N}$, and $\eta_{S}^{N}$ were extracted for each animal (n = 9), and the yield ratios corresponding to Metric 4 were computed. One mouse used for protocol optimization was excluded. Across animals, $\eta_{L}^{T}$ consistently exceeded $\eta_{S}^{T}$ (Fig. 7b), indicating a dominant contribution of the long-FLT component within tumor volumes. In normal liver (Fig. 7c), $\eta_{S}^{N}$ generally exceeded $\eta_{L}^{N}$, although two mice showed elevated $\eta_{L}^{N}$ consistent with a $\tau_{L}-\tau_{S}$ crosstalk observed *in situ*. The yield ratio derived from Metric 4 showed a strong and statistically significant correlation with WB-measured PD-L1 expression (r^2^ = 0.77, p < 0.01; Fig. 7d). These results demonstrate that tomographic fluorescence yield analysis provides a robust *in vivo* metric that reflects PD-L1 expression in deep-seated orthotopic HCC tumors.

## Discussions

Noninvasive molecular imaging methods that quantify receptor expression *in vivo* are essential for preclinical drug development and for clinical applications such as patient stratification for targeted therapies and longitudinal monitoring of treatment response without repeated biopsies. Considerable progress has been made in developing biomarker-targeted imaging agents, including antibodies, nanobodies, peptides, and engineered scaffold proteins, along with radio-labeling and optical labeling strategies for PET and fluorescence imaging^55–58^. A persistent limitation across these approaches is that targeted probes exhibit varying degrees of nonspecific accumulation within the TME, while conventional imaging methods primarily report total probe uptake^59,60^. As a result, these approaches cannot distinguish between probes that are specifically associated with the intended molecular target and nonspecifically retained probes within the TME, limiting the accuracy of receptor quantification.

Prior work from our group and others has shown that FLT measurements of targeted NIR probes can reveal distinct FLT components within tumors^42,45,50^. These components may reflect differences in molecular interactions between the probes and their target biomarkers. This observation provides a potential strategy for improving molecular specificity by separating fluorescence signals based on FLT rather than intensity alone. We have previously shown that *in vivo* FLT imaging enables accurate quantification of tumor biomarkers such as EGFR and PD-L1 in superficial tumors^45,51^. Building on this foundation, we establish in this study a comprehensive ATD-FLT tomography approach to quantify PD-L1 expression in orthotopic hepatocellular carcinoma (HCC) models. By integrating *in situ* FLT imaging, decay amplitude estimation, and ATD-FLT tomography, we established a quantitative metric that correlates strongly with *ex vivo* PD-L1 expression. This metric accounts for both nonspecific probe accumulation in the liver and crosstalk between long- and short-FLT components, enabling accurate quantification across tumors with substantial baseline heterogeneity. Across both *in situ* imaging (Fig. 5) and tomographic reconstruction (Fig. 7), the same normalization strategy yielded consistent correlations with Western blot measurements (r^2^ = 0.77), supporting the robustness of this approach across imaging geometries.

Systematic evaluation of the four amplitude-based metrics highlighted the importance of normalization strategies that incorporate both nonspecific probe retention and $\tau_{L}-\tau_{S}$ crosstalk. While Metrics 1 and 2 showed poor agreement with WB, Metric 3 showed a moderate positive correlation with PD-L1 expression (Fig. 5e, r^2^ = 0.63), indicating that normalizing long FLT amplitudes from tumors $\left(a_{L}^{T}\right)$ by total probe uptake in normal liver $\left(a_{L}^{N}+a_{S}^{N}\right)$ is more appropriate than using only the short FLT amplitudes from tumor ($a_{S}^{T}$, Metric 1) or normal liver ($a_{S}^{N}$, Metric 2). A key observation was the presence of a measurable long-FLT component in normal liver $\left(a_{L}^{N}\right)$, despite the lack of PD-L1 expression, indicating crosstalk between the predefined FLT components ($\tau_{L}$ and $\tau_{S}$). Correcting for this effect by subtracting the crosstalk $\left(a_{L}^{N}\right)$ from $a_{L}^{T}$ (Metric 4) produced the strongest correlation with PD-L1 expression (Fig. 7d, r^2^ = 0.77, p < 0.01), establishing it as the most reliable parameter for biomarker quantification in deep-seated orthotopic HCC tumors.

Interestingly, differences were observed between reflectance-based *in situ* imaging and *in vivo* tomographic measurements. *In situ* measurements frequently (7 out of 10 tumors) showed dominance of the short-FLT component $\left(a_{S}^{T}>a_{L}^{T}\right)$ within tumors (Fig. 5a), whereas tomographic reconstruction yielded higher contributions from the long-FLT component across tumor volumes $\left(\eta_{L}^{T}\right.>\eta_{S}^{T})$ (Fig. 7b). This discrepancy likely reflects intrinsic differences in the sampling depth of the two imaging modalities. Reflectance imaging is dominated by signals arising from superficial tissue layers, where hepatic arterial perfusion is greatest and nonspecific probe accumulation is therefore highest^61^. In contrast, tomographic imaging integrates fluorescence originating from the full depth of the liver, including deeper regions with lower arterial flow, which may result in reduced nonspecific probe uptake. Comparison with our prior work in orthotopic breast tumor models further underscores the importance of considering the anatomical site of the tumor^51^. Metrics such as mean FLT and amplitude ratios such as Metric 1 that performed well in breast tumors did not generalize to the liver, where nonspecific uptake and $\tau_{L}-\tau_{S}$ crosstalk are more pronounced. These findings highlight the need for tissue-specific normalization strategies and suggest that similar considerations may apply to other clearance organs (e.g., kidneys).

In this study, the long $\left(\tau_{L}\right)$ and short $\left(\tau_{S}\right)$ FLT components were determined based on the FLT distributions across the normal liver and HCC from *in situ* FLT measurements. We hypothesize that the long and short FLT components reflect distinct molecular binding states, including potential interactions with PD-L1 expressing cells or nonspecific retention within extracellular compartments. This assumption is supported by extensive prior work from our group and others demonstrating increased FLTs of biomarker targeted NIR fluorescent probes^42,45,51^. However, direct attribution of these FLT components to specific molecular states will require further investigation. Future studies using intravital fluorescence lifetime imaging microscopy (FLIM) at cellular resolution will be critical to directly map FLT variations to probe localization and microenvironmental context within the TME. Our strategy for estimating $\tau_{L}$ and $\tau_{S}$ assumes that tumor pixels with the longest FLTs predominantly contain PD-L1 associated αPDL1-800, whereas pixels within normal liver, which lacks PD-L1 expression, primarily reflect the nonspecifically retained αPDL1-800 fraction. Using the *in situ* determined $\tau_{L}$ and $\tau_{S}$ allowed us to fit only the asymptotic portion of the decay curves rather than performing bi-exponential fitting of the full TD decay data. This approach is particularly advantageous because recovering all four parameters ($\tau_{L},a_{L},\tau_{S}$, and $a_{S}$) from bi-exponential fits is a fundamentally ill-posed, nonlinear problem prone to degeneracy and noise artifacts^48^. Fixing $\tau_{L}$ and $\tau_{S}$ converts the amplitude recovery into a linear problem, substantially reducing susceptibility to noise and degeneracy. Prior studies have shown that by pre-determining either FLTs or amplitudes enhances the accuracy of bi-exponential decay parameter estimation^51^. Recent advances in fit-free machine learning approaches enable extraction of all four decay parameters from bi-exponential TD data, offering promising alternatives when pre-determining FLT components is not feasible^62,63^. Collectively, these complementary strategies expand the toolkit for reliable multi-component FLT analysis in complex biological systems.

Compared with previous optical imaging and tomography studies, which have largely focused on improving spatial resolution or imaging depth^64–66^, this work establishes and validates a quantitative crosstalk-corrected metric for molecular biomarker estimation in deep-seated tumors using ATD-FLT tomography. The ATD approach exploits late arriving photons (asymptotic regime), where the diffusion approximation is valid, enabling accurate three-dimensional reconstruction of fluorescence yield distributions^54^. Prior reports have shown that ATD-FLT tomography provides accurate recovery of fluorophore distributions when FLTs exceed the intrinsic diffusion timescale of tissue (~0.4 ns)^54^, a condition well-satisfied by αPDL1-800. Here, we show that the ATD approach is well suited for resolving the three-dimensional distribution of distinct αPDL1-800 FLT populations, represented by the long $\left(\eta_{L}\right)$ and short $\left(\eta_{S}\right)$ components *in vivo*, particularly in clearance organs such as the liver where nonspecific probe uptake is substantial.

Future work will focus on expanding this approach to larger animal cohorts and additional tumor models to define sensitivity limits for PD-L1 quantification and assess generalizability. Extending this framework to other immune biomarkers and therapeutic targets will further evaluate its broader applicability. The need for such validation is reinforced by the substantial baseline heterogeneity in PD-L1 expression that we observed even among tumors generated under identical conditions. Furthermore, PD-L1 levels may differ markedly between primary and secondary lesions within the same subject^67,68^. Such intrinsic heterogeneity presents a major challenge in preclinical therapeutic studies, where variability in baseline immune biomarker expression levels can obscure true treatment effects. The ability to noninvasively quantify biomarker expression in individual lesions offers a practical advantage by enabling stratification based on baseline molecular heterogeneity, reducing variability and improving study design. Future studies will incorporate immunotherapy models to determine how these FLT-derived metrics relate to treatment response and dynamic changes in the TME.

In summary, this study demonstrates that normalization of fluorescence decay amplitudes, combined with correction for FLT crosstalk, enables ATD-FLT tomography to quantitatively assess PD-L1 expression in deep-seated orthotopic HCC tumors, despite substantial baseline PD-L1 heterogeneity. By resolving distinct FLT components across the full tumor volume, this approach provides a robust metric for quantifying immune markers across subjects and establishes a strong foundation for future studies aimed at tracking immunotherapy response. Although optical imaging depth is inherently limited, diffuse optical tomography has already demonstrated feasibility in human breast cancer^69^, and the growing availability of clinically relevant fluorescent targeting agents positions this methodology as a timely and complementary advance in molecular imaging^70,71^.

## Supplementary Material

Supplementary Files

This is a list of supplementary files associated with this preprint. Click to download.

- Supplementarymaterials.docx
